# “Somewhere I belong?” A study on transnational identity shifts caused by “double stigmatization” among Chinese international student returnees during COVID-19 through the lens of mindsponge mechanism

**DOI:** 10.3389/fpsyg.2022.1018843

**Published:** 2022-10-18

**Authors:** Ruining Jin, Xiao Wang

**Affiliations:** ^1^Civil, Commercial and Economic Law School, China University of Political Science and Law, Beijing, China; ^2^Suzhou Lunhua Education Group, Suzhou, China

**Keywords:** transnational identity, double stigmatization, Chinese international students, returnees, political othering, social stigma, mindsponge mechanism

## Abstract

Chinese international students who studied in the United States received “double stigmatization” from American and Chinese authorities because of the “political othering” tactic during COVID-19. The research used a phenomenological approach to examine why and how specifically the transnational identity of Chinese international students in the United States shifted during the double stigmatization. The researcher conducted a total of three rounds of interviews with 15 Chinese international students who studied in the United States and returned to China between 2018 and 2020, which culminated in 45 interviews through a longitudinal study to probe the transnational identities of this population before and during the double stigmatization; the study also examined how the mindsponge mechanism worked during the identity shifts and the interplay among stigmatization, transnational identity shifts, and the mindsponge mechanism. The study concluded that before COVID-19, Chinese international students had been stigmatized in both China and the United States. And there were three identity clusters for international students’ transnational identity: homestayers, wayfarers, and navigators based on four dimensions: intercultural competence, relocation of locality, diaspora consciousness, and attachment between China and the US. The study concluded that during the double stigmatization, Chinese international students in all three identity clusters took individualism into their core values, whereas Chinese traditional values, such as nationalism, collectivism, and obedience to authority waned. In addition, the study corroborated the trust evaluator’s gatekeeper role and substantiated the validity and effectiveness of cost-benefit analysis on an individual’s decision to accept or reject new information and values.

## Introduction

The term “transnational identity” generally applies to people who have cross-cultural living experiences between the home country and the host countries (Esteban-Guitart and Vila, 2015). Such a transnational identity is common among international students who pursue professional training and academic degrees overseas. Students with transnational identities have an in-and-between cognitive intimacy between themselves and others when talking about their home and host country (Wang, 2022). However, the formation of transnational identity is not an instant process; rather, according to the mindsponge mechanism, it involves a gradual and incessant information processing that international students undertake to either accept or reject new information and values to acculturate to the current living environment (Vuong and Napier, 2015). During this process, international students’ in-and-between cognitive intimacy between home and the host country will be shifted accordingly depending on how much information and values the students choose to accept/reject.

In the past decade, China has become a major source for outputting international students to study in foreign countries (QS, 2020), and the outbreak of COVID-19 in January 2020 had led to the mask-wearing practice among this large population in the United States, for which they were stigmatized by US politicians and government agencies (Ma and Zhan, 2022). Because of the COVID-19 safety concerns and mask-wearing stigmatization, as the leading source of international students in the US, the Chinese international student population plunged in the academic year 2020–2021 after a decade-long growth, from 372,532 in 2019 to 317,229 in 2020 (Statista, 2022). However, what coincided with their return was the blame game between China and the US, and their identity as returnees from the United States had made them caught in the crossfire in domestic anti-America sentiment (Yu, 2021). As a result, returnees experienced “double stigmatization”: the initial stigmatization was imposed by the US government and the Centers for Disease Control and Prevention (CDC)’s mask mandating rule, whereas the subsequent stigmatization stemmed from Chinese domestic anti-west populism/cyber-nationalism (Yu, 2021). A few studies have been conducted on Chinese international students’ stigmatization abroad in western countries such as the US, the UK, and their social media (Roberto et al., 2020; Catalano and Wang, 2021; Smith et al., 2022); yet limited research has been done in terms of domestic stigmatization occurred during the COVID-19 and how the double stigmatizations shifted these returnees’ transnational identity gradually. Therefore, the research aims to answer the following research questions,

1. How were Chinese international students’ transnational identities before COVID-19? How did the double stigmatization affect their identity between China and the US?
2. How did the mindsponge mechanism work during the identity shifts? And during these identity shifts, what new values were acquired and gradually became their core values, and what previous core values waned to become peripheral or rejected?
3. What is the interplay among stigmatization, transnational identity shifts, and the mindsponge mechanism?

## Background

### COVID-19 and political othering

COVID-19 is named after the coronavirus pandemics that broke out in December 2019 in the epicenter of Wuhan, China. It is caused by severe acute respiratory syndrome coronavirus 2 (SARS-CoV-2). A Public Health Emergency was issued by the World Health Organization to warn people across the globe of the severity of the pandemics. As of May 2022, it has infected 528 million people globally, killing 68 million (World Health Organization [WHO], 2020).

Political othering is a term to describe the tactic that demagogues generally adopt to consolidate their right-wing/far-right populist voter base (Wodak, 2020). In this discourse, “us” will be described in a positive light and differentiated from “they” or “them,” which are usually accompanied by negative traits (Catalano and Wang, 2021). In the case of the COVID-19 blame game, both the US and Chinese officials adopted political othering.

### Trump’s China rhetoric, policies, and the first stigmatization during the COVID-19

Facing the COVID-19 pandemic, former President of the United States Donald J. Trump stigmatized and politically othered China and Chinese international students by accusing the Chinese government of mishandling and the spread of COVID-19. He also embraced the conspiracy theory that COVID-19 was a bioweapon developed by China (Okediya, 2020). The accusation and anti-China rhetoric had led to a chain of hate crimes and Sinophobia in the United States, as Chinese immigrants and international students were found to be the victims of this racism-driven discrimination and crimes (Okediya, 2020; Costello et al., 2021; Ma and Miller, 2021; Xu et al., 2021). Turning accusations and rhetoric into policies, in May 2020, Trump issued his executive order *Proclamation on the Suspension of Entry as Nonimmigrants of Certain Students and Researchers from the People’s Republic of China*, asserting that Chinese international students were political operatives of the People’s Republic of China (PRC) who engaged in a broad range operation as an attempt to obtain technologies and intellectual property belonging to the United States (United States, Executive Office of the President [Donald Trump], 2020). This executive order would impact thousands of Chinese international students, and many Chinese international students would consider other destinations such as the UK and Australia (Weinstein and Peterson, 2020).

The impacts of the first stigmatization on Chinese international students have been discussed in previous studies (Ma and Miller, 2021; Costello et al., 2021; Xu et al., 2021, Ma and Zhan, 2022), and it was confirmed that the mask-wearing in this population had triggered racist encounters and their mask-wearing practice had been stigmatized by the American government and CDC. Specifically, by comparing the American government’s polarized stance on mask-wearing in 2 months, Ma and Zhan (2022) illustrated how the American government orchestrated and manipulated the stigma of mask-wearing on Chinese international students and then how they reversed the rhetoric to make the mask-wearing a mandate and destigmatize the same practice. Other studies have also indicated that social stigma was likewise imposed on Chinese overseas students (Balingue, 2021; Xu et al., 2021).

### China’s reaction and soaring nationalism

While being finger pointed by many countries for the origin and mishandling of COVID-19, China adopted a similar, if not identical tactic—by counteraccusations and counter-conspiracy theories directed toward the US, China also used the political othering tactic to rally domestic support in the name of nationalism. For example, the Foreign Ministry spokesman Hua Chunying had repeatedly hinted at the correlation between the virus outbreak and the US biological lab at Fort Detrick to counter the US’s Wuhan Biolab Leak conspiracy theory (Cheng et al., 2022). In this context, anti-west and anti-US sentiment had been fueled during COVID-19 in Chinese society and social media (Catalano and Wang, 2021) to establish a “binary opposition” among netizens to coerce public figures with transnational identity or cosmopolitanism to take a stand between the China US and America Others (Tao, 2021, p. 634). For example, the Chinese controversial writer Fang Fang was stigmatized by netizens for her critical view of China in her published diary, where she recorded the mishandling of the local government (Tao, 2021).

## Double stigmatization

In addition to Fang Fang and public figures, other populations with a transnational identity or cosmopolitan views were also forced to take a stand during this bilateral tension, including Chinese international student returnees who were once again stigmatized during their reintegration into Chinese society. During an encounter with local medical personnel in her hotel quarantine, a returnee demanded bottled water but was rejected, which the returnee considered a violation of her human right (Global Times, 2020). The video clip of this altercation went viral on Chinese social media, and Chinese netizens overwhelmingly sided with the medical personnel and stigmatized the returnee as a “giant infant” for defying COVID-19 prevention measures (Yu, 2021). Soon the stigmatization was escalated to a higher level across the country when a state-run TV station host commented on the returnee’s return as a malicious attempt to poison the motherland (Joe, 2020). By doing so, the authority harnessed cyber-nationalism to increase domestic cohesion and discredit western education as a “giant infants” producer and those who represented western values as “giant infants.”

## Theoretical framework

### Transnational identity and its variations

Transnational identity is characterized by four distinct features: intercultural competence, reconstruction of locality, diaspora consciousness, and mixed senses of belonging (Wang, 2022). Intercultural competence refers to language, cultural knowledge, and awareness that one possesses which helps to bridge the gap interculturally; reconstruction of locality refers to the (re)creation of social spaces transnationally (Tedeschi et al., 2020). Diaspora consciousness refers to “a broader and critical reflection toward the US and Others” (Wang, 2022, p. 4); lastly, the mixed senses of belonging, refers to where an individual would draw the line between US and Others (Wang, 2022). Furthermore, Wang (2022) coined the term “Variations of Transnational Identity” and categorized Chinese students’ transnational identity variations into three identity clusters: the “Homestayers” who stand firmly with the Chinese US, the “Wayfarers” who prefer the lifestyle of foreign Others, and the “Navigators” who maneuver between the Chinese US and foreign Others (p. 16). The homestayer identity cluster is marked by those students who demonstrate a high affinity with the Chinese US and a low affinity to the foreign Others, along with their lack of interest and willingness to explore the Others’ culture and values; the wayfarer identity cluster, however, suggests individuals who belong to this cluster would alienate their Chinese US and view China critically and cynically, while embracing values and cultures in the foreign US to be socially embedded in the host country; the third identity cluster, the navigator identity, is defined by members’ flexibility and maneuverability to navigate through various emotional attachments, acclimatize to different social norms and values, and make flexible strategies to accommodate transnational lives (Wang, 2022).

This categorization is in accordance with Sussman (2010)’s three types of return identity findings, in which attached, detached, and universal orientation was identified as three identities based on their extent of attachment toward their countries of origin. Moreover, Peng (2015) asserted that returnees navigate in a dynamic and interconnected way, where such an identity is relative, temporally, and situationally reactive. The variations of transnational identity serves as a theoretical base for the research to compare and contrast the self-formation of the returnees’ transnational identity before and after the two massive stigmatizations and see how it would impact the dynamics of the transnational identities.

## Mindsponge mechanism

**FIGURE 1:**
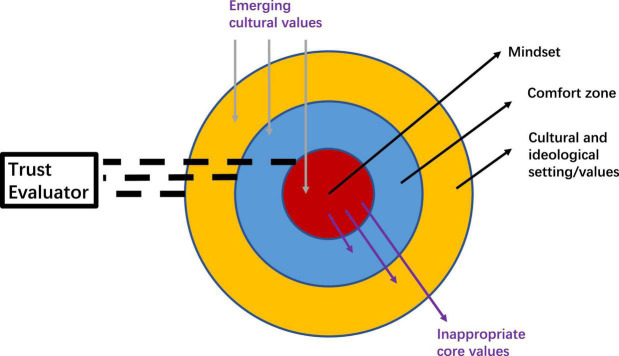
A modified version of mindsponge mechanism (Vuong and Napier, 2015).

Coined by Vuong and Napier (2015), the term mindsponge mechanism describes how an individual absorbs and integrates new cultural values into one’s own set of core values. Overall, the mechanism centers on five major components: the mindset, comfort zone, multi-filtering system, cultural and ideological setting, and cultural values (see Figure 1).

According to Vuong and Napier (2015), the mindset is a reflection of one’s core values and is represented by the red nucleus in the center of the diagram, so when evaluating emerging cultural values or information from outside, one would compare the usefulness and appropriateness of the new ones with the existing mindset. Based on compatibility, one would then make the decision to accept or reject them.

Enclosing the mindset, the comfort zone is the blue circle that represents values and information that are supportive or compatible with one’s mindset. According to Vuong and Napier (2015), the comfort zone serves as a buffering zone to protect one’s mindset from the impacts of information or values that are different from one’s core value and helps to filter the emerging value and information’s acceptability. Based on references from the mindset, the 3D multi-filtering system evaluates information as it enters the mind. The outcome of the filtering process is the decision either to accept the information into the mindset, toss it out, or keep it in the comfort zone for later evaluation.

The filtering process involves one’s cost-benefit analyses and trust evaluation. Cost-benefit analyses weigh information differently based on their values by relating and comparing it to the references kept in the mindset as well as other information from the outside world and one’s comfort zone. Moreover, trust evaluators function as the conductor who checks the “tickets” (validity) of new information and grant trusted information a “priority pass” so that it can be quickly accepted without going through the typical full evaluating process. However, when information is distrusted, it will also receive an expedited rejection.

### Stigma as a social construct

The earliest definition of stigma could be traced back to the last 60 s when Canadian sociologist Erving Goffman coined the term stigma as an attribute about a person or a group that is deeply discredited and rejected by society (Goffman, 1963). Later, Kleinman and Hall-Clifford (2009) expanded Goffman’s definition of stigma from a psychological perspective to a sociopsychological perspective that centers on the “social, economic, and political” aspects that shape “the distribution of stigma within a social milieu” (p. 2). In its own words, it asserted that stigma should be viewed as a social construct, as “the stigmatized and those who stigmatize are interconnected through local social networks. Although stigma may share features across contexts, it uniquely affects lives in local contexts” (p. 2).

Link and Phelan (2001) delineated the critical function of stigma—to distinguish “US” from “Them” (p. 370). By listing a series of stigmatization in the history of the United States, the author articulated how politics came to play when it comes to telling differences between the outgroups “Them” and the ingroup “US” (p. 370). The study further explained the process of stigmatization, that once the differences are established, stereotyping would follow-up to further discredit the stigmatized. Eventually, *via* US vs. Them narrative, politicians could “bond the authorities with the public, stressing that they are standing with and representing their people” so that by using the concept of US, they can increase the domestic cohesion and “gain public support and power resources” (Cheng et al., 2022).

## Empirical approach

This research study used a phenomenological approach in an attempt to answer the research questions:

1. How were Chinese international students’ transnational identities before COVID-19? How did the double stigmatization affect their identity between China and the US?
2. How did the mindsponge mechanism work during the identity shifts? And during these identity shifts, what new values were acquired and gradually became their core values, and what previous core values waned to become peripheral or rejected?
3. What is the interplay among stigmatization, transnational identity shifts, and the mindsponge mechanism?

## Research design

According to Creswell and Poth (2016), a population needs to be identified to study the research problem. In this case, the population was all Chinese students who studied in the United States and returned to China after the outbreak of the COVID-19 pandemic. The target population was all Chinese returnees (including student visa holders, green-card holders as well as work visa holders) from the U.S. post–COVID-19. Although it is controversial to specify the exact sample size in general for qualitative research (Dworkin, 2012), “there is indeed variability in what is suggested as a minimum. An extremely large number of articles, book chapters, and books recommend guidance and suggest anywhere from 5 to 50 participants as adequate” (p. 1). Given that Chinese international students are a highly homogenous group, the study interviewed 15 participants through a purposive sampling technique. According to Tongco (2007), the purposive sampling technique, composed of a common probability sampling technique, is a non-probability sampling that is “most effective when one needs to study a certain cultural domain with experts within” (Tongco, 2007, p. 1). The researcher used the purposive sampling technique and conducted a total of three rounds of interviews with 15 Chinese international students who studied in the United States and returned to China between 2018 and 2020 (see Table 1 for participants’ demographics), which culminated in 45 interviews through a longitudinal study. The first round of interviews was conducted on 22–24 December 2018. The second round of interviews was conducted on 25–27 January 2020. The third round of interviews was conducted on 17–19 April 2020. The researcher conducted all the interviews through the WeChat application’s video chatting function. Through various channels, such as Chinese International Student Associations, social networks, cultural activities, and job affairs, the researchers recruited the participants when they were college students in the United States before the first round of interviews. Since human subjects were involved, the study was reviewed and approved by Institutional Review Board (IRB).

**TABLE 1:** Participants’ demographics.

Age	Gender	Length of stay (years)	Area of study in the US	Expertise	Funding sources
22	M	4	Business	Admin	Public
22	F	5	Business	Accounting	Self-funded
24	F	2	Business	Financing	Self-funded
30	F	11	Education	Education leadership and policy	Self-funded
28	M	2	Business	Accounting	Self-funded
25	M	3	Business	Administration	Scholarship
26	F	2	Education	Math teaching	Self-funded
27	F	6	Computer programing	–	Scholarship
26	M	2	Computer programing	–	Self-funded
26	F	3	Business	Admin	Self-funded
25	M	6	Business	Project management	Self-funded
26	F	3	Business	Public relation management	Self-funded
27	F	2	Business	Advertising	Self-funded
26	M	2	Business	Human resources management	Self-funded
25	M	5	Business	Logistic management	Self-funded

### Interview questions

On a scale of 0 to 5 (0 as not true/none, and 5 as extremely true/very much), participants were individually asked to answer five essential questions in three rounds and given additional time for articulation and experience-sharing. Each question has two subthemes asking if they are more likely to happen in China or America. The first question, which aims to address intercultural competence, is about if they are confident to make sense in a cross-cultural setting and get things done, with options to give different scores to express their confidence level from 0 to 5 in China and America, respectively. Then the second question is to address the reconstruction of locality; therefore, the question is that on a scale of 0–5, how much social capital/networks do they think they possess in China and America, respectively? The third question is about diaspora consciousness, as the researchers asked the participants on a scale of 0–5, how many social norms and values from China and America they uphold, respectively. Fourth, the question is straightforward: on a scale of 0–5, evaluate the attachment to China and the United States. Lastly, the questions examine the magnitude of stigma one feels on a scale of 0–5 in China and the US, respectively.

### Data

The researchers took notes and used audio recordings during the interview. Recordings were transferred to use a professional transcribing device and labeled participants in pseudonym names to ensure confidentiality. Transcripts were checked and proofread and double-checked with the participant to make sure that the transcribed information is of one’s true intent. After the coding, the researcher drew tables to categorize and summarize related concepts, themes, and subthemes. Then the researchers established a table for each participant through a radar chart to visualize the data.

## Findings

In the section that follows, the findings are organized under three themes: (1) transnational identity among different identity clusters before COVID-19; (2) identity shifts after the mask-wearing stigmatization; and (3) identity shifts after the double stigmatization.

### Cluster criteria

The three identity clusters are listed as homestayers (abbreviated as H marked by the green lines); wayfarers (abbreviated as W with the purple lines); and Navigators (abbreviated as N and marked by red lines). The last question—stigmatization is not related to one’s transnational identity; therefore, the answer value is excluded from the cluster assigning criteria. In the first four questions, in this China vs. the US narrative, if one’s China scores outweigh the US in three or more interview questions, one will be assigned to the homestayer cluster; if one’s US scores outweigh the China ones, one will be assigned into wayfarer cluster; the rest will be assigned into the navigator cluster.

### Transnational identity among different identity clusters before COVID-19

A minority of participants in this group (3 out of 15) were identified as homestayers before COVID-19, as they reported strong emotional attachments to China and embraced Chinese values and practices when living and studying in the US. They answered questions in Chinese, and all acknowledged that their English and cultural proficiency was not proficient enough to build and sustain social networks with local people. They reported a strong sense of being stigmatized (5 out of 5) in the US society even before COVID-19. As homestayers, they adhered to Chinese tradition and discarded the influence of US cultures and ways of life during the 3D multiple filtering process, as they were non-compatible with homestayers’ previous core values. In other words, a large amount of information and values during their study in the US before COVID-19 could not even make it to their comfort zone before being repelled as inappropriate core values. Rebecca, a 24-years-old graduate student, shared her typical example of being in the identity cluster and the rejection of US influence.

Although I’m in the United States, I always watch Youku dramas and use WeChat to socialize with my domestic friends and family. I don’t have many US friends, because I think we are not on the same page, and they cannot get my point and know what I’m thinking about. I tried to use Facebook, Twitter, and Instagram at the very beginning, but when I established my accounts on these platforms and posted my selfies, people were messaging me like, “you looking for a sugar daddy? How much you ask for?” I was so pissed off by harassment like this and I think this is a typical stereotype that many have toward Chinese female students.

Two participants in this group (2 out of 15) were identified as wayfarer identity, as their responses indicated a stronger preference for the US over China in this US vs. Them narrative. Overall, their English proficiency, especially oral English proficiency was above average for international students based on the interview performances, and they were adventurous in exploring the US society and culture and expanding their US social networks. In general, they displayed a stronger attachment to the US and were critical of China. Unlike homestayers, the wayfarers generally accepted and embraced changes around them, along with the information and values they received from the US studying and living experience, as this new information and values were in alignment with the identity cluster’s definitions and their mindsets. Some of the information and values made it to the comfort zone, while others even reached the core. However, reversely, although wayfarers’ mindset also kept a portion of the core values of the past, a certain amount of the core values had already been ejected to the comfort zone or even deemed as inappropriate values. William explained his stated:

I love freedom and this is what it is supposed to be. Back home (in China) you cannot be your true self and speak up against certain evil and malice because of you know, political correctness. I really love being who I am here in the States. But I do celebrate holidays such as Spring Festival and Moon Festival though. To better engage in the local community, I gotta do more things that people do here, so I made lots of new friends but because I barely keep myself updated with my domestic friends, say I no longer play the same mobile phone game we did back in China, so I kinda feel the gap is gettering bigger between me and my domestic friends. Lastly, I think discrimination is a universal phenomenon, and the difference is, that back home we discriminate against each other based on region and social status, whereas here it’s more racially biased.

Third, the navigators are versatile warriors that prevailed in almost all aspects of this matrix and kept a balance between China and the US. A majority of participants (10 out of 15) were categorized into this identity cluster, in which they could make adaptions accordingly to fit into different social norms and values in both countries and indicated a high level of confidence in their international competitiveness. They demonstrated an emotional attachment to both countries, and they built and sustained social networks in their new surroundings. In other words, while temporarily integrating new information and values into their comfort zone, navigators did not let their core values wane compared to that of the wayfarers. However, stigmatization was also felt in both countries. Jeff recalled his transnational stories as follows:

I’m okay with people here and there and I love them all, I mean you just do different things with different people, that’s it. Here in the States, I hang out with my local friends in the dog run, talking about pets, National Football League (NFL), Major League Baseball (MLB), things you know, the American guys are interested in. Back home to get me and my friends connected, we play pc games that are not ping-sensitive, so that it could be a fair game even though I’m in the States, connecting to the game server from afar. I feel attached to both countries, and I celebrate important festivals of both countries, too. But speaking of stigmatization, I would say stuff like that happens here and there, too. I have a White American girlfriend, but when my Chinese classmates saw me and her together, many would stare at us, and some would later come to me and say, “hey buddy you must be rich.” And when I posted our photos on my Chinese social media account, many questioned that the reason why I chose to be with her was to get a green card.

Overall, homestayers and wayfarers tend to have polarized answers to most questions and are located at two opposite ends between China and the US. The outcomes of the filtering process to either accept, reject, or put new information and values in the comfort zone also varied based on the three different identity clusters accordingly, as homestayers tend to reject these changes; wayfarers demonstrated a stronger willingness to integrate them, and navigators were more likely to keep new information and values into a comfort zone for later consideration. There were a few convergences of two identity clusters somewhere in the figure, but no rally point was found to unite the three identity clusters except stigmatizations from the US subtheme, where each scored 5, 5, and 3, respectively.

### Transnational identity during the COVID-19 mask-wearing

Compared with their Figure 2 responses, the cultural competence and relocation of locality did not show a significant change in Figure 3. But all participants expressed a stronger sense of stigma in the US due to the mask-wearing practice. While homestayers mostly described their stigmatization experience based solely on their mask-wearing practice, wayfarers and navigators were confronted in a stereotypical and micro-aggressive way due to cultural misconception and misinformation. Notably, during this time for navigators, even though they kept a certain amount of the old core values, they were gradually allowing more newly acquired information and values to be in the comfort zone, if not reaching the core. Chris—a navigator, described his feelings of being stigmatized during this social interaction:

**FIGURE 2:**
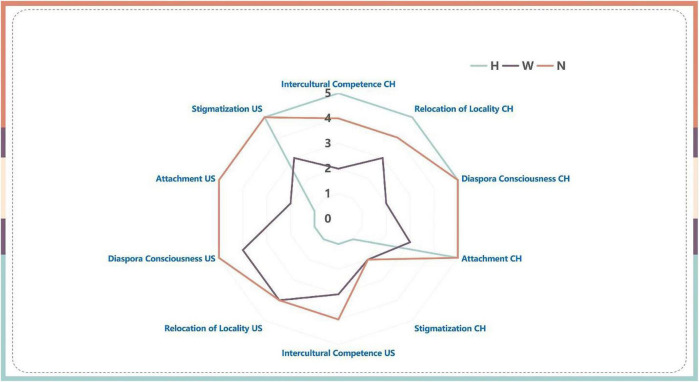
Three identity clusters before COVID-19.

**FIGURE 3:**
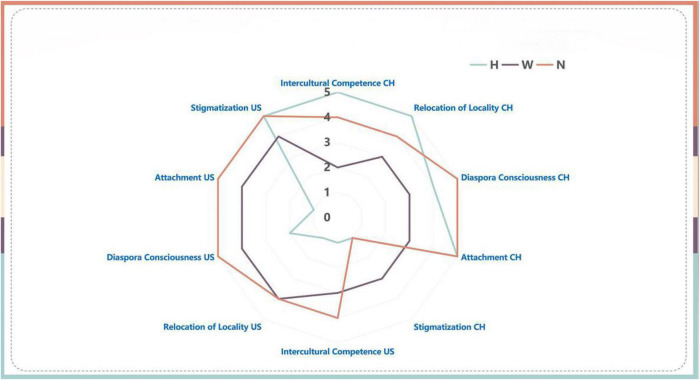
Identity shifts of the three identity clusters during the mask-wearing.

Once I was dining with my friends in a Chinese restaurant, I did not wear a face mask so I wouldn’t be considered “different” from others, but somehow when we were looking at the menu, a friend asked the waiter, “where is barbecue bat? This is a Chinese restaurant, and you don’t have that?” I was offended and teased back, “do you have a pistol in your pocket? Aren’t you an American?”

Other similar incidents occurred through interactions in academic settings and daily life, including wayfarers—the identity clusters that indicated a strong willingness to be socially embedded in America. The wayfarers during this stage continued to allow new information and values to be in the comfort zone and the core, while pushing out the core values from the past mindset. Steve recalled his frustration and stigmatization as follows:

I disobeyed my parents and lied to them that I would wear a mask every day when I went outside, but actually, I did not do that. I mean, I speak standard English with a North American Accent, I love cheeseburgers and I party hard. But when it comes to masking wearing, there are still people who keep asking. “Hey, why aren’t you wearing masks as other Asians do?” I think regardless of what I do and how desperately I wish I could fit in here, they still treat me like aliens. And that is very disheartening.

However, a majority of the participants admitted that their stigmatization encounters that occurred during COVID-19 were mostly about mask-wearing practices, especially for homestayers. Rejecting the new information and values in the US (the mask-wearing stigmatization), the homestayers stuck to the influence and way of life in China and kept mask-wearing a daily practice. And for this reason, they were further stigmatized. Constantine, a 22-years-old homestayer who reported his stigmatized, if not racist experience when he was using public transport:

One time when I was on a bus and wore a mask, other passengers stared at me and would rather stand far away from me than sit next to me. I felt myself a monster in the eyes of the beholders. And then one senior lady yelled at me from afar, “why are you here? You should take off your masks, or go back to China and eat the bat, you China virus!” I was very dismayed by the intended alienation and bat-eating stereotypes.

Notably, stigmatizations did not all come from Americans, some of the stigmatizations came from their Chinese peers. Sandy as a navigator complained about her embarrassment during a lecture class:

During a lecture class, there was a Chinese girl who abruptly jumped onto the podium, saying that she apologized to everyone because of COVID-19 and that the Chinese ate everything they saw so there came the virus. This is absurd, by now there wasn’t any substantial evidence to suggest that COVID-19 originated from China. I totally disagree with the girl and the apologetic culture she stands for.

To sum up, participants’ transnational identity shifts during this time were marked by intensified feelings of being stigmatized in the US. Although their attachment to both countries and diaspora consciousness went through some fluctuations, in general, there were no significant changes to dramatically change their identity cluster from one to another. However, as homestayers repeated their previous decision to reject new information and values from the US, navigators and wayfarers began to intake a greater amount of new information and values to their comfort zone and core values.

### Double stigmatizations and the identity shifts

**FIGURE 4:**
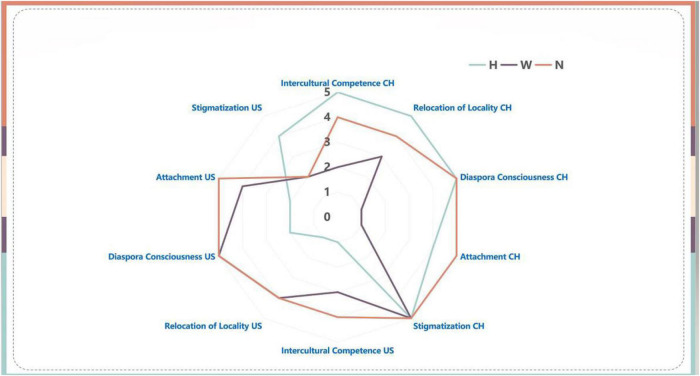
Identity shifts of the three identity clusters after the double stigmatizations.

Eventually, after three rounds of interviews, there was a rallying point in Figure 4 for all identity clusters: the stigmatization all participants felt during the bottled water incident and the media’s campaign to stigmatize Chinese returnees. Participants in all three identity clusters expressed their grievance about being returnee students, and an understanding, if not full support to the international student who demanded bottled water. Compared with the resistance displayed in the previous interview, homestayers by the time they returned to China, had demonstrated a minor extent of change in their mindset, namely a greater concern for individualist pursuits. Abby is a homestayer, and she voiced her opinion:

When I was in the US, I thought I was different from this society and saw China as my home where I felt my heart truly belongs. But we were paying a high price for coming back home and obeying the shelter-at-place quarantine policy. Since we are paying money to stay in a motel-like hotel, I mean honestly, I’ve never stayed in any hotels below four stars before this, so we have a right to basic services like water and food. I could imagine if the water wasn’t purified, a kettle won’t help at all. The video clip didn’t disclose the full picture in terms of how bad the water quality was from the hotel, but netizens and TV hosts jumped in to condemn us for our characteristics of “disobeying.”

As wayfarers furthered down their path to embrace more information and values from the US, their core values were mainly defined by US liberal and universalistic values, such as freedom of speech and political activism. Therefore, they became more cynical and critical of a more authoritative Chinese society and COVID-19 prevention measures. As a wayfarer, Steve spoke up against the “injustice” and “unfair treatment” he received upon returning:

A Chinese domestic streaming platform abruptly shut my streaming while I was talking about how the quarantine policy and Zero-COVID could be political because industries that could be operated remotely such as IT, gaming, sourcing, and things like that would not be severely impacted by the COVID-19 lockdowns, but to those less skilled, labor-intensive industries where jobs could not be done remotely, they will lose more if they fully comply with the lockdown policy. So, there should have been some reimbursement policies or financial incentives directed toward these industries, or there are no obligations for companies in these industries to uphold the rules of Zero-COVID policy.” Then I was cut off and I was warned by the censor manager that I must show full support to the anti-COVID measures and policies, or my streaming account would be suspended permanently. I tried to argue and reason, but he just said that I know you back from the States, this is China so you gotta know the “Chinese Characteristics” or don’t come back if you act like a “giant infant” like the bottled water princess.

For navigators, although the extent to which they allowed the US acquired information and values to enter their core values differed person-by-person, they generally demonstrated a greater extent of demand for individual rights, transparency, and authority’s responsibility. Sally, a navigator, cast her doubt on hotel charges and the services it offered during the quarantine and the stigmatized experience she encountered.

The hotel charged me about 600 Y=(about 100$) per night, which is supposed to be a 5-star hotel level, but everything here sucks, no bottled water, no heated food, unstable internet, and bed bugs. When I questioned the hotel manager, he responded, “you paid hundreds of thousands for the return ticket and now you care about several hundred (RMB)?” When I argued for transparency and responsibility, he responded, “you second-rich (refers to children of first-generation rich) ask for so many, you should at least celebrate for being alive. If you don’t want to stay here, go back to where you came from.” The humiliation and stigma were so much that if there were 10 points for this question, I would give it a 10!

Overall, the double stigmatization, although did not lead to any defection of participants from one identity cluster to another, somehow rallied all participants for the first time in terms of stigmatization felt. By this time, all three identity clusters had embraced a different level of values from the US, thus showing various extents of dissatisfied responses to the second stigmatization.

## Discussion and conclusion

### Discussion of RQ1

This study examines the transnational identity of Chinese international students who studied in the United States before and during COVID-19, and how the double stigmatizations impacted their identity shifts. The findings provide theoretical and empirical knowledge to existing literature regarding the interplay and dynamics between transnational identity shifts and sociopolitical activities and practices. The study has demonstrated that before COVID-19, Chinese international students had been stigmatized in both China and the United States. And there were three identity clusters for international students’ transnational identity: homestayers, wayfarers, and navigators based on four dimensions: intercultural competence, relocation of locality, diaspora consciousness, and attachment between China and the US.

This study offers new empirical evidence regarding how stigma as a social construct is being used by authorities and politicians to alienate and stigmatize some and unite others as a power play to consolidate their power. The differences are, in the United States, mask-wearing social stigma resulted from Trump Administration’s policies and CDC guidance, while in China the returnee-shaming stemmed from social media campaigns and state-run media’s propaganda efforts.

The study exemplifies that the political othering technique could be implemented through stigmatization. The double stigmatization serves as both sides’ attempt to differentiate “US” from “Them” in sociopolitical discourses. During COVID-19, both the US and China had associated negative attributes with Chinese international students and thus stigmatized this population to consolidate a better image of “US.”

Furthermore, the study suggested that although Chinese international student returnees encountered double stigmatizations, there were no defections in the identity clusters of their transnational identity. For instance, a homestayer would not change one’s attachment even though one indicated a stronger magnitude of stigmatization from the China side than on the US side. Likewise, a wayfarer would remain in their identity cluster during and after the mask-wearing stigma.

### Discussion of RQ2

The study concluded that during the double stigmatization, in general, Chinese international students in all three identity clusters took individualism into their core values, as they all called for human rights protection in the case of “bottled water incident.” Wayfarers and navigators further incorporated more liberal western values such as political activism, freedom of speech, and called for government responsibility when they cast doubt on the legitimacy of the COVID-19 prevention measures and the shelter-in-place order. Moreover, Chinese traditional values, such as nationalism, collectivism, and obedience to authority waned, as displayed by navigators and wayfarers who defied Chinese authority and their parents’ suggestion for mask-wearing, and the display of all three identity clusters’ grievances during the second stigmatization.

The study corroborates the trust evaluator’s gatekeeper role from the mindsponge mechanism, that when the source of the information and value is trusted, these information and values will be given a “priority pass,” and vice versa, they will be rejected quickly. In this case, because wayfarers had preference and trust in the US, they accepted information and values from the US in an expedited way. For example, accepting the information and moving it to the core, the two wayfarers in this study embraced the social norms in the US and did not wear a mask; and the unnamed apologetic Chinese international student who jumped on the podium and apologized for COVID-19 during a class also selectively believed in the pro-US conspiracy theory that COVID-19 was made in China, while rejecting China’s counteraccusations because of the distrust toward Chinese authority.

The study also substantiated the validity and effectiveness of cost-benefit analyses on an individual’s decision to accept or reject new information and values from the mindsponge mechanism. For example, initially, Rebecca created her social media accounts in the US, because she had accounts in Chinese social media, so her social media account creation in the US is compatible with the mindset, and therefore through the 3D multiple filtering process, such a behavior, along with the social media use in the US were placed into the comfort zone based on the compatibility with her mindset. However, her decision to discard the use of Facebook, Instagram, and Twitter was after the cost and benefit analyses—that her social circle was mainly composed of domestic friends whom she could only virtually hangout with through Chinese social media, and that her feedback from US social media was mostly negative. As the costs outweighed the benefits, Rebecca decided to quit using US social media and kick it out as an inappropriate value.

### Discussion for RQ3

The interplay among stigmatization, transnational identity shifts, and the mindsponge mechanism is a complicated process. First of all, a social construct as previously mentioned, stigmatization functions to differentiate the in-group from the out-group. Once the two groups were formed in the US, Chinese international students with different transnational identities had to pick a side. For those who chose the US side, like the wayfarers, some of the navigators, and the apologetic Chinese student, their trust evaluator was turned on to grant a “priority pass” to information and values from the US. In this case, they all complied with the social norms in the US and took off their masks to be more American. Some even joined Trump to question China and apologize for the Chinese mishandling of the pandemic; and for those who stood with the China side, stigmatization became a sign of distrust that homestayers and some of the navigators would use as a reference to expedite the rejection process for US information and values.

During the “bottled water incident,” the identical pick-one dilemma occurred and these students with different transnational identities were forced to decide between being a “giant infant” who indulged in individualistic pursuits and a collectivism-driven person who sacrificed their own interests for the big picture of COVID-19 prevention. In short, the social stigmatization would amplify the magnitude of trust evaluator in the 3D multiple filtering process of the mindsponge mechanism. Once the trust evaluator is turned on, it would give priority pass or denial to new information based on the source. Because of this, an expedited acceptance or denial of information would promote more identity formations of wayfarers and homestayers who hold polarized views on many fronts, while impeding the identity formation of navigators who hold a balanced view between the two countries.

## Implications, limitations, and future studies

The study corroborated existing literature regarding the correlation between Chinese international students’ English proficiency and their social integration in English-speaking host countries. For instance, the wayfarers who indicated a stronger willingness and motivation to be socially embedded into the US society generally had a higher language proficiency than homestayers who demonstrated a greater China root. However, because the scope of this study is narrowed down to the COVID-19 double stigmatization-related identity shifts of the returned international students, the researchers failed to identify language proficiency measurements, and what specifically caused the reintegration hardship of wayfarers back in China and why their social integration in the home country was lower than the host country. A possible approach to examine the issue in future studies might include the use of an expanded version of the theoretical framework—Mindsponge Theory in probing human social behaviors among this identity cluster (Vuong, 2022).

Furthermore, there could be an additional identity cluster to the existing homestayer, wayfarer, and navigator classification, should one indicate a low achievement in intercultural competence, an inability to create social networks in both places, a low level of cultural and value preservation from both countries, and a low attachment to both China and the US. Future studies examining the demographics and the formation of this identity cluster could be done based on Bayesian Mindsponge Framework analytics—an innovative method for social and psychological research (Nguyen et al., 2022).

## Data availability statement

The original contributions presented in this study are included in the article/supplementary material, further inquiries can be directed to the corresponding author.

## Ethics statement

The studies involving human participants were reviewed and approved by the Institutional Review Board at China University of Political Science and Law. The patients/participants provided their written informed consent to participate in this study.

## Author contributions

RJ: conceived and designed the analysis, performed the analysis, wrote the manuscript, and final approval of the version to be published. XW: wrote the manuscript, interpreted the Chinese literature for the work, and revised the work critically for important intellectual content. Both authors contributed to the article and approved the submitted version.
